# Antidepressant intervention to possibly delay disease progression and frailty in elderly idiopathic pulmonary fibrosis patients: a clinical trial

**DOI:** 10.1007/s40520-025-03009-4

**Published:** 2025-03-22

**Authors:** Hongyan Ren, Zheng Wang, Yafen Jiang, Qing Mu, Yaxin Li, Jing Wang, Tiantian Cui, Qijie Sun, Xiaojv Zhang

**Affiliations:** Department of Respiratory and Critical Care Medicine, Zhengzhou University People’s Hospital, Henan Provincial People’s Hospital, No. 7, Weiwu Road, Jinshui District, Zhengzhou, 450003 Henan Province China

**Keywords:** Depression, Elderly, Frailty, Idiopathic pulmonary fibrosis, Lung function

## Abstract

**Background:**

Idiopathic pulmonary fibrosis (IPF) is more likely to occur in the elderly population, and these patients often become depressed. It has been recognized that psychological disorders are not conducive to the control of many diseases. Thus, this study aims to determine whether alleviating depression can delay the progression of IPF and frailty in elderly patients with IPF.

**Methods:**

IPF patients over 60 years old were included in the study. None had a prior history of psychological disorders. All developed depression after being diagnosed with IPF. During the 12-month follow-up, some patients received anti-depression interventions and the rest didn’t. Depression, IPF, frailty and peripheral inflammation at baseline and after follow-up were evaluated by indicators and scales such as BDI-II, FVC %pred, 6MWT, mMRC, CFS, TFI, SGRQ, K-BILD, IL-6, and TNF-α. Multivariate logistic regression was employed for data analysis.

**Results:**

There were 213 elderly patients with IPF. Among the 89 patients who received anti-depression interventions, the above-mentioned indicators and scales did not deteriorate during the follow-up period (P > 0.05). Among the remaining 124 patients, the FVC %pred, and 6MWT levels decreased, and the mMRC grade, CFS, TFI, SGRQ and K-BILD scores, and peripheral IL-6 and TNF-α levels increased during the follow-up period (P < 0.05).

**Discussion:**

Compared with non-intervened IPF patients, those receiving anti-depression interventions seemed to maintain a certain stability in IPF, frailty, and peripheral inflammation over a period.

**Conclusion:**

Improving depression may help delay the deterioration of patients' IPF and frailty at certain stages.

Trial registration: Registration on UMIN-CTR. Registration number: UMIN000057161. Date of registration: February 27th, 2025.

**Supplementary Information:**

The online version contains supplementary material available at 10.1007/s40520-025-03009-4.

## Introduction

Idiopathic pulmonary fibrosis (IPF) is a chronic interstitial lung disease of unknown cause that commonly occurs in the elderly population. In recent years, epidemiological data shows that there are more than 500,000 new IPF cases worldwide each year, and the incidence rate is still increasing at a rate of about 1.9% annually [1]. This disease can severely damage lung function, and subsequently have a wide-reaching impact on tissues and organs throughout the body [2]. More significantly, the progression of this disease is almost irreversible, with a poor prognosis [3]. Currently, the median survival period of IPF patients is only 2–5 years [4]. So, this disease poses a serious threat to human health, especially to the health of the elderly, and it is necessary to enhance interventions at multiple levels.

Three recent cross-sectional studies reported that the prevalence of depression among patients with IPF had increased significantly, and the degree of depression was related to the condition of the disease and the patients' quality of life [5–7]. Currently, it is evident that there are sufficient reasons to believe that suffering from IPF can significantly increase the incidence of depression. However, it remains unclear whether psychological disorders such as depression can, in turn, affect the progression and prognosis of IPF. Certainly, there is some theoretical basis for this reverse effect. (1) Psychological disorders can trigger autonomic nervous system imbalance, causing airway constriction, changes in pulmonary blood flow, and aggravating pulmonary hypoxia. (2) Psychological disorders can lead to an increase in pro-inflammatory factors, inhibit anti-fibrotic factors, and exacerbate the process of pulmonary fibrosis. (3) Psychological disorders can cause patients to develop unhealthy lifestyles and reduce patients’ compliance, thus affecting the treatment effect. Some previous studies have also confirmed that depression is related to abnormal pulmonary function indexes in the general population [8, 9]. So, it’s quite possible that improving the psychological state of IPF patients has some positive impact on disease progression and prognosis, but this is still rather speculative.

Frailty is a clinical syndrome closely associated with aging. In patients with IPF, due to the influence of age and the disease itself, this clinical syndrome is very common and poses significant harm [10]. Additionally, there seems to be a complex connection between frailty and psychological disorders. Previous studies at the phenotypic and genotypic levels have shown a bidirectional influence between frailty and depression [11–13]. That is, in IPF patients, improving the psychological state might have a protective effect on frailty and ultimately contribute to improving the patients' prognosis.

**Fig. 1:**
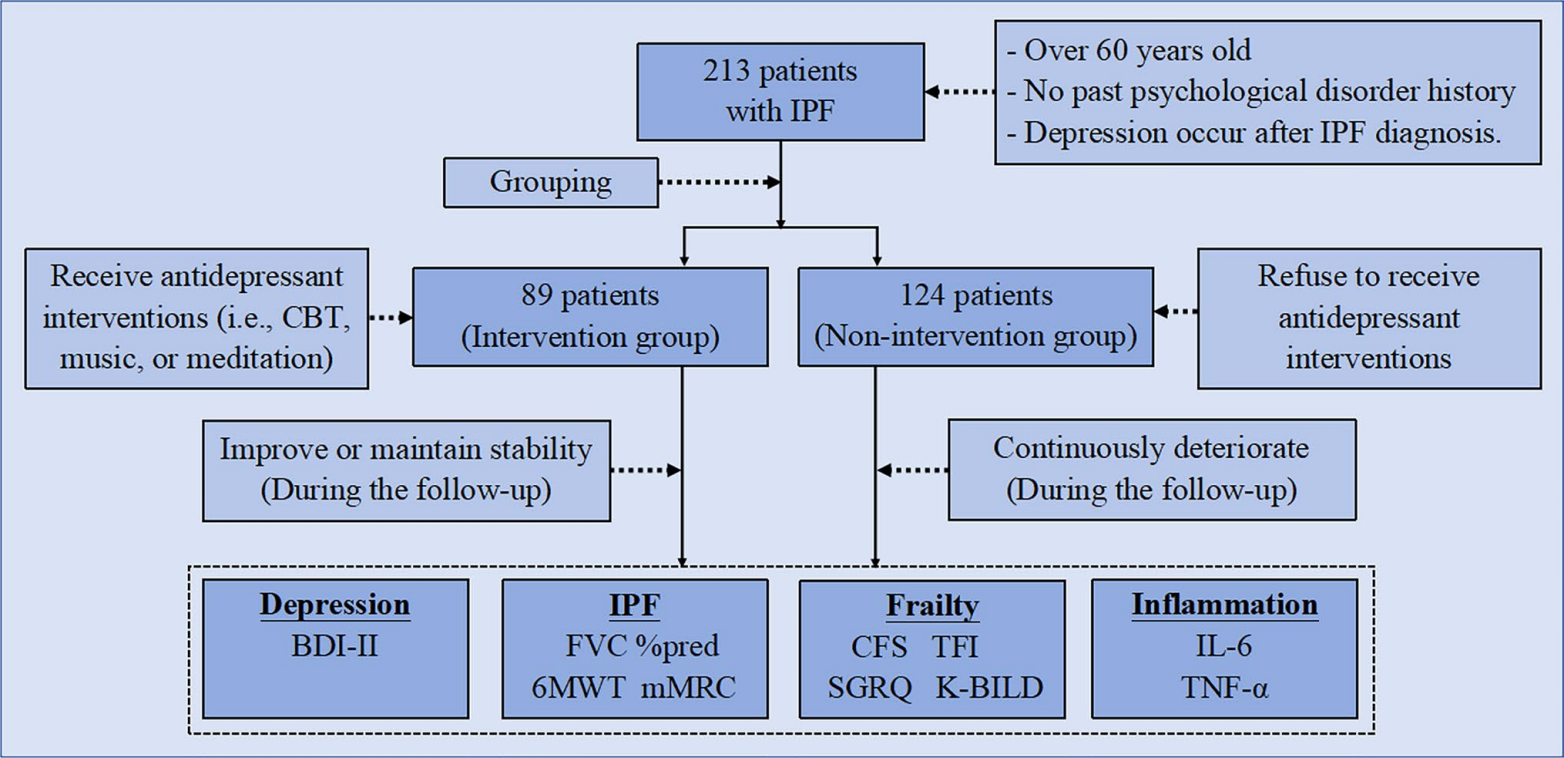
Flow Diagram of This Study. IPF = Idiopathic pulmonary fibrosis, CBT = Cognitive behavioral therapy, BDI-II = Beck Depression Inventory-II, FVC %pred = Forced vital capacity percent predicted, 6MWT = 6-min walk test, mMRC = modified Medical Research Council dyspnea scale, CFS = Clinical Frailty Scale, TFI = Tilburg Frailty Indicator, SGRQ = St. George's Respiratory Questionnaire, K-BILD = King’s Brief Interstitial Lung Disease questionnaire, IL-6 = Interleukin-6, TNF-α = Tumor necrosis factor-α

It should be emphasized that the psychological state is unlikely to be the decisive factor influencing the progression and prognosis of IPF. However, the role of the psychological state in this regard should not be completely overlooked, which is in line with the spirit of the bio-psycho-social medical model. Therefore, this study included more than 200 elderly IPF patients with secondary depression, aiming to explore whether intervening in depression can help delay the exacerbation of IPF and frailty. The flow diagram of this study was shown in Fig. 1. The results obtained will help us understand the potential role of psychological factors in the progression and prognosis of IPF and provide a basis for developing an intervention measure for elderly IPF patients.

## Materials and methods

### Ethics requirement

This study received approval from the Medical Ethics Committee of Henan Provincial People's Hospital. Given that the research subjects were patients with IPF, the study was conducted in strict compliance with the stipulations of the World Medical Association's Declaration of Helsinki. Subsequently, on February 27th, 2025, a clinical trial registration was carried out on UMIN-CTR, with the registration number being UMIN000057161. Moreover, all patients and their families consented to participate in the study and signed the informed consent forms.

### Subject recruitment

A total of 213 elderly patients with IPF who were treated at Henan Provincial People’s Hospital from January 1, 2018 to December 31, 2022 were consecutively included. The inclusion criteria were as follows: (1) Age ≥ 60 years old. (2) According to the ATS/ERS/JRS/ALAT clinical practice guidelines, all patients were diagnosed with IPF [14]. Briefly, high-resolution CT showed the pattern of usual interstitial pneumonia, while excluding the influence of pathogenic factors such as the environment, drugs, and connective tissue diseases. (3) There was no previous history of any mental or psychological disorders, that is, there had never been any symptoms related to mental or psychological illnesses that lasted continuously for more than 24 h, and there was no relevant medical treatment experience either. (4) After the diagnosis of IPF, different degrees of low mood, decreased interest, and anhedonia gradually appeared (lasting for more than two weeks), and at the time of this admission, depression was present as assessed by the Beck Depression Inventory-II (BDI-II) (total score ≥ 14) [15]. (5) After being diagnosed with IPF, the patients survived for at least one year and were followed up during this period. (6) No history of any malignant tumors. (7) The conditions of common chronic geriatric diseases were stable.

### Intervention and grouping

All patients were advised by their attending physicians to receive intervention measures for treating depression. Patients had the freedom to choose to receive intervention at this hospital or other hospitals, or at counseling institutions or community centers. The optional intervention methods included cognitive behavioral therapy (CBT), music therapy, and meditation. The treatment courses were generally between 8 and 20 sessions, and each session was approximately 45–60 min. After completing the above treatment course, the attending physician also recommended that the patient continue the intervention in this regard on their own after returning home. It should be especially emphasized that the intervention methods and treatment courses were determined by the patients or the intervention institutions they chose. The researchers did not interfere in any way, but merely recorded information related to the psychological intervention afterwards.

In the end, 89 patients received the intervention measures for depression, while the other 124 patients refused. In the data analysis of this study, they were classified into the intervention group and the non-intervention group respectively.

In addition, all patients received conventional anti-IPF treatment, including symptomatic therapy, anti-fibrotic therapy, and rehabilitation therapy.

### Baseline data collection

The baseline characteristics of patients, such as gender, age, smoking history, drinking history, body mass index (BMI), and history of chronic diseases, were obtained from medical records.

As mentioned previously, depression was assessed using the BDI-II [15]. This scale consists of 21 items that evaluate various aspects of depressive symptoms. Each item is scored on a scale from 0 to 3. A total score between 0 and 13 usually indicates normal, between 14 and 19 indicates mild depression, between 20 and 28 indicates moderate depression, and between 29 and 63 indicates severe depression.

The time from the diagnosis of IPF to the current admission was recorded. The forced vital capacity percent predicted (FVC %pred) and the diffusing capacity of the lung for carbon monoxide percent predicted (DLCO %pred) were used to evaluate lung function. The 6-min walk test (6MWT) and the modified Medical Research Council dyspnea scale (mMRC) were used to assess the symptom burden of IPF in patients. Among them, the mMRC is a tool for assessing the severity of dyspnea [16]. It is divided into five levels from 0 to 4, representing mild to extremely severe dyspnea respectively.

The Clinical Frailty Scale (CFS) and the Tilburg Frailty Indicator (TFI) were used to assess the frailty of the patients. The St. George's Respiratory Questionnaire (SGRQ) and the King's Brief Interstitial Lung Disease questionnaire (K-BILD) were used to evaluate the quality of life of patients with IPF and to assist in the assessment of frailty.

The CFS rates an individual's frailty level on a scale from 1 (very fit) to 9 (terminally ill) by considering factors such as mobility, independence in daily life, and overall health [17]. The TFI, on the other hand, evaluates frailty from three dimensions: physical, psychological, and social functions. It consists of 15 items with a total score of 15 [18]. A score of 0–4 indicates non-frailty, 5–8 indicates pre-frailty, and 9–15 indicates frailty. The SGRQ is composed of three parts: symptoms, activities, and impacts, with a total score ranging from 0 to 100 [19]. The higher the score, the greater the impact of the disease on the patient's respiratory health status and quality of life. The K-BILD covers aspects such as the degree of dyspnea, limitations in daily activities, psychological state, and awareness of the disease [20]. In this study, the most commonly used version in China was adopted. Its total score also ranges from 0 to 100. The higher the score, the more significant the impact of the lung disease on the patient's quality of life.

### Follow-up

Using the above-mentioned methods, the depression, IPF, and frailty of all patients were also evaluated 12 months after enrollment.

### Peripheral inflammation detection

3 ml of blood was collected from each patient at admission and after 12 months respectively. The samples were subjected to routine centrifugation to collect plasma samples, which were then stored at – 70 ℃. Commercial ELISA kits were used to detect the levels of peripheral interleukin-6 (IL-6) and tumor necrosis factor-α (TNF-α) (ID: EZIL6, EZHTNFA-150 K; Sigma-Aldrich). All procedures were completed in accordance with the manufacturer's instructions.

### Statistical analysis

Continuous variables were expressed as mean ± standard deviation. The differences between groups for these variables were evaluated by independent sample t-test, and the t-value and P-value were reported. Categorical variables were expressed as frequency (proportion). The differences between groups for these variables were evaluated by chi-square test, and the chi-square value and P-value were reported. The differences between groups for these variables were also analyzed by multivariate logistic regression, and the odds ratio (OR), 95% confidence interval (95% CI) and P-value were reported. This multivariate model adjusted for the basic characteristics of patients and also adjusted for the above indicators of depression, IPF, frailty, and peripheral inflammation. A P value less than 0.05 indicated that the difference or correlation was statistically significant.

## Results

### Baseline characteristics

In Table 1, compared with the non-intervention group, the proportions of patients with type 2 diabetes mellitus and coronary heart disease were significantly higher in the intervention group (P = 0.032, P = 0.030). In addition, there were no significant differences between the two groups in terms of gender, age, smoking history, drinking history, BMI levels, and other chronic disease histories (P > 0.05).

**Table 1:** Baseline characteristics in the two groups

	Intervention	Non-intervention	t / χ^2^ value	P value
Total (n)	89 (100.0)	124 (100.0)	–	–
Gender (n)				
Male	68 (76.4)	90 (72.6)	0.396	0.529
Female	21 (23.6)	34 (27.4)		
Age (years)	78.1 ± 9.0	79.4 ± 7.7	1.081	0.281
Smoking (n)	62 (69.7)	76 (61.3)	1.592	0.207
Drinking (n)	22 (24.7)	19 (15.3)	2.943	0.086
BMI (kg/m^2^)	24.3 ± 2.9	23.7 ± 2.9	1.503	0.134
Chronic diseases (n)				
T2DM (n)	17 (19.1)	40 (32.3)	4.576	0.032
HTN (n)	35 (39.3)	59 (47.6)	1.432	0.231
CHD (n)	15 (16.9)	37 (29.8)	4.734	0.030
CVD (n)	8 (9.0)	17 (13.7)	1.115	0.291
COPD (n)	7 (7.9)	13 (10.5)	0.418	0.518
Asthma (n)	6 (6.7)	10 (8.1)	0.131	0.718

Continuous variables were presented as mean ± standard deviation, and the differences between groups were evaluated by independent sample t-test. Categorical variables were presented as frequency (proportion), and the differences between groups were evaluated by chi-square test. P < 0.05 indicated statistically significant differences

*BMI* Body mass index, *T2DM* = Type 2 diabetes mellitus, *HTN* = Hypertension, *CHD* = Coronary heart disease, *CVD* = Cerebrovascular disease, *COPD* = Chronic obstructive pulmonary disease

### Baseline depression, IPF, frailty and peripheral inflammation

In Supplemental Table 1, at baseline, there was no difference in the BDI-II score and the number of patients with mild, moderate or severe depression between the intervention group and the non-intervention group (P > 0.05). There was no difference in the FVC %pred, DLCO %pred, 6MWT, mMRC grade, CFS, TFI, SGRQ and K-BILD scores between the two groups (P > 0.05). There was no difference in the peripheral IL-6 and TNF-α between the two groups (P > 0.05). However, the time from diagnosis of IPF to this admission in the intervention group was significantly longer than that in the non-intervention group (P = 0.001).

### Interventions and treatments during the follow-up period

In Table 2, 61, 28, and 18 patients in the intervention group received CBT, music therapy, and meditation treatment respectively. The treatment courses were 12.9 ± 3.4, 15.1 ± 3.0, and 16.1 ± 2.7 sessions respectively. Among these patients, 49 received the above-mentioned treatments in major hospitals, while 30 and 10 received them in counseling institutions and community centers respectively. In addition, the patients in both groups received symptomatic drugs, antifibrotic drugs and/or rehabilitation therapy according to their actual conditions, and there was no significant difference between the two groups in the types of these anti-IPF treatment (P > 0.05).

**Table 2:** Interventions and treatments during the follow-up period in the two groups

	Intervention	Non-intervention	t/χ^2^ value	P value
Antidepressant treatment (n)				
CBT	61 (68.5)	–	–	–
Music	28 (31.5)	–	–	–
Meditation	18 (20.2)	–	–	–
Treatment courses (n)				
CBT	12.9 ± 3.4	–	–	–
music	15.1 ± 3.0	–	–	–
meditation	16.1 ± 2.7	–	–	–
Antidepressant institution (n)				
Major hospitals	49 (55.1)	–	–	–
Counseling institutions	30 (33.7)	–	–	–
Community centers	10 (11.2)	–	–	–
Anti-IPF treatment				
Symptomatic drugs (n)	81 (91.0)	117 (94.4)	0.885	0.347
Antifibrotic drugs (n)	7 (7.9)	13 (10.5)	0.418	0.518
Rehabilitation (n)	24 (27.0)	23 (18.5)	2.135	0.144

CBT = Cognitive behavioral therapy, CT = Course of treatment, IPF = Idiopathic pulmonary fibrosis. Continuous variables were presented as mean ± standard deviation, and the differences between groups were evaluated by independent sample t-test. Categorical variables were presented as frequency (proportion), and the differences between groups were evaluated by chi-square test. P < 0.05 indicated statistically significant differences

### Differences in depression, IPF, frailty and peripheral inflammation between baseline and after follow-up

In Table 3, in the intervention group, the BDI-II score after follow-up was significantly lower than that at baseline (P = 0.004). However, there was no difference in the FVC %pred, 6MWT, mMRC, CFS, TFI, SGRQ and K-BILD scores, peripheral IL-6 and TNF-α levels between baseline and after follow-up (P > 0.05). Only the DLCO %pred level was significantly lower after follow-up than at baseline (P = 0.038).

**Table 3:** Differences in depression, idiopathic pulmonary fibrosis, frailty and peripheral inflammation between baseline and after follow-up in the two groups

Intervention group	After follow-up (After 12 months)	Baseline (On admission)	t/χ^2^ value	P value
Total (n)	89 (100.0)	89 (100.0)	–	–
Depression				
BDI-II	20.9 ± 5.7	23.5 ± 6.5	2.893	0.004
IPF				
FVC %pred	73.7 ± 12.1	76.2 ± 11.7	1.373	0.172
DLCO %pred	48.0 ± 11.3	51.3 ± 9.8	2.089	0.038
6MWT	398.6 ± 45.7	396.2 ± 41.8	0.354	0.724
mMRC	2.3 ± 1.0	2.1 ± 0.9	1.437	0.152
Frailty				
CFS	4.5 ± 1.4	4.3 ± 1.2	1.414	0.159
TFI	8.0 ± 2.2	7.5 ± 2.1	1.622	0.107
SGRQ	43.8 ± 15.5	41.9 ± 14.5	0.848	0.397
K-BILD	72.7 ± 16.0	69.4 ± 13.7	1.458	0.147
Peripheral inflammation				
IL-6 (pg/ml)	17.68 ± 4.54	18.42 ± 4.18	1.119	0.265
TNF-α (pg/ml)	37.05 ± 12.41	35.05 ± 12.04	1.092	0.276

Non-intervention group	After follow-up (After 12 months)	Baseline (On admission)	t/χ^2^ value	P value
Total (n)	124 (100.0)	124 (100.0)	–	–
Depression				
BDI-II	25.4 ± 6.8	23.3 ± 6.7	2.537	0.012
IPF				
FVC %pred	71.0 ± 12.5	78.2 ± 12.2	4.577	< 0.001
DLCO %pred	47.2 ± 9.7	52.5 ± 9.8	4.328	< 0.001
6MWT	375.8 ± 50.6	393.0 ± 51.2	2.657	0.008
mMRC	2.4 ± 1.2	1.9 ± 1.2	3.408	0.001
Frailty				
CFS	5.5 ± 1.5	4.1 ± 1.2	8.666	< 0.001
TFI	9.2 ± 2.4	7.2 ± 2.0	7.317	< 0.001
SGRQ	45.2 ± 18.1	38.6 ± 15.9	3.069	0.002
K-BILD	82.0 ± 19.5	70.6 ± 15.3	5.136	< 0.001
Peripheral inflammation				
IL-6 (pg/ml)	19.65 ± 5.56	17.88 ± 4.57	2.738	0.007
TNF-α (pg/ml)	42.48 ± 11.51	36.00 ± 12.83	4.188	< 0.001

Continuous variables were presented as mean ± standard deviation, and the differences between groups were evaluated by independent sample t-test. Categorical variables were presented as frequency (proportion), and the differences between groups were evaluated by chi-square test. P < 0.05 indicated statistically significant differences.

*BDI-II* Beck Depression Inventory-II, *IPF* Idiopathic pulmonary fibrosis, *FVC %pred* Forced vital capacity percent predicted, *DLCO %pred* Diffusing capacity of the lung for carbon monoxide percent predicted, *6MWT* 6-min walk test, *mMRC* modified Medical Research Council dyspnea scale, *CFS* Clinical Frailty Scale, *TFI* Tilburg Frailty Indicator, *SGRQ* St. George’s Respiratory Questionnaire, *K-BILD* King’s Brief Interstitial Lung Disease questionnaire, *IL-6* Interleukin-6, *TNF-α* Tumor necrosis factor-α.

In Table 3, in the non-intervention group, the BDI-II score, mMRC grade, CFS, TFI, SGRQ and K-BILD scores, peripheral IL-6 and TNF-α levels were significantly higher after follow-up than at baseline (P = 0.012, P = 0.001, P < 0.001, P < 0.001, P = 0.002, P < 0.001, P = 0.007, P < 0.001), while the FVC %pred, DLCO %pred and 6MWT levels were significantly lower after follow-up than at baseline (P < 0.001, P < 0.001, P = 0.008).

### Differences in depression, IPF, frailty and peripheral inflammation between baseline and after follow-up assessed by multivariate logistic analysis

In Table 4, the multivariate model showed that in the intervention group, the BDI-II score and DLCO %pred level of the subjects significantly decreased during follow-up (OR = 0.362, 95% CI = 0.206–0.635, P < 0.001; OR = 0.716, 95% CI = 0.538–0.953, P = 0.022), while there were no significant changes in other IPF, frailty, and peripheral inflammatory indicators (P > 0.05).

**Table 4:** Differences in depression, idiopathic pulmonary fibrosis, frailty and peripheral inflammation between baseline and after follow-up in the two groups assessed by multivariate logistic regression

Baseline vs after follow-up	Intervention group		Non-intervention group	
	OR (95% CI)	P value	OR (95%CI)	P value
Depression				
BDI-II	0.362 (0.206–0.635)	< 0.001	1.884 (1.199–2.961)	0.006
IPF				
FVC %pred	0.864 (0.680–1.097)	0.229	0.635 (0.515–0.783)	< 0.001
DLCO %pred	0.716 (0.538–0.953)	0.022	0.604 (0.468–0.780)	< 0.001
6MWT	0.782 (0.422–1.450)	0.435	0.540 (0.339–0.861)	0.010
mMRC	1.246 (0.921–1.685)	0.153	1.436 (1.157–1.781)	0.001
Frailty				
CFS	1.175 (0.939–1.472)	0.159	2.248 (1.781–2.839)	< 0.001
TFI	1.119 (0.976–1.284)	0.107	1.507 (1.323–1.717)	< 0.001
SGRQ	1.107 (0.912–1.343)	0.305	1.278 (1.103–1.479)	0.001
K-BILD	1.186 (0.974–1.443)	0.089	1.425 (1.226–1.657)	< 0.001
Peripheral inflammation				
IL-6	0.905 (0.664–1.234)	0.529	1.070 (1.019–1.125)	0.007
TNF-α	1.014 (0.989–1.038)	0.275	1.044 (1.022–1.067)	< 0.001

The multivariate model was adjusted for the basic characteristics of patients and also adjusted for the above indicators of depression, idiopathic pulmonary fibrosis, frailty, and peripheral inflammation. P < 0.05 indicated statistically significant differences

*BDI-II* beck depression inventory-II, *IPF* Idiopathic pulmonary fibrosis, *FVC %pred* Forced vital capacity percent predicted, *DLCO %pred* Diffusing capacity of the lung for carbon monoxide percent predicted, *6MWT* 6-min walk test, *mMRC* modified medical research council dyspnea scale, *CFS* clinical frailty scale, *TFI* Tilburg frailty indicator, *SGRQ* St. George’s respiratory questionnaire, *K-BILD* King’s brief interstitial lung disease questionnaire, *IL-6* Interleukin-6, *TNF-α* Tumor necrosis factor-α, *OR* odds ratio, *95% CI* 95% confidence interval

In Table 4, the multivariate model also showed that in the non-intervention group, the BDI-II level of the subjects significantly increased during follow-up (OR = 1.884, 95% CI = 1.199–2.961, P = 0.006), the FVC %pred, DLCO %pred and 6MWT levels significantly decreased during follow-up (OR = 0.635, 95% CI = 0.515–0.783, P < 0.001; OR = 0.604, 95% CI = 0.468–0.780, P < 0.001; OR = 0.540, 95%CI = 0.339 ~ 0.861, P = 0.010), and the mMRC grade, CFS, TFI, SGRQ and K-BILD scores, peripheral IL-6 and TNF-α levels significantly elevated during follow-up (OR = 1.436, 95% CI = 1.157–1.781, P = 0.001; OR = 2.248, 95% CI = 1.781–2.839, P < 0.001; OR = 1.507, 95% CI = 1.323–1.717, P < 0.001; OR = 1.278, 95% CI = 1.103–1.479, P = 0.001; OR = 1.425, 95% CI = 1.226–1.657, P < 0.001; OR = 1.070, 95% CI = 1.019–1.125, P = 0.007; OR = 1.044, 95% CI = 1.022–1.067, P < 0.001).

## Discussion

Under the bio-psycho-social medical model, the impact of psychological factors on diseases and health is garnering more and more attention [21]. Patients with IPF are prone to developing psychological disorders like depression [5–7]. Against this backdrop, this study boldly explored whether intervening in depression can offer some degree of protection to the IPF and frailty. Given the limited treatment options for IPF and their subpar efficacy, this research topic holds considerable practical significance. The research results obtained may introduce a new, low-cost and easily-achievable approach to the intervention of IPF in the elderly population.

According to the analysis of baseline data, this study confirmed that there was no difference in depression, IPF, frailty, peripheral inflammation, and most of the basic characteristics of the subjects between the two groups. For a few research variables such as the time from IPF diagnosis to this admission, although the distribution was unbalanced between the groups, the degree was acceptable and could be adjusted by subsequent multivariate analysis. So, the quality of the baseline data in this study was satisfactory, making the results after 12 months of follow-up highly comparable, which increased the reliability of this study.

During the follow-up period, patients in the intervention group independently received intervention treatment for depression. The researchers neither participated in the selection of hospitals or institutions nor in the formulation of intervention plans. Instead, they only recorded information as observers. The intervention methods received by the patients, i.e., the three mentioned earlier, were all jointly determined by the relevant hospitals or institutions and the patients. These methods are characterized by their simplicity of implementation. They neither increase the economic and energy burden on patients nor pose additional health risks to them [22–24]. In addition, most patients in both groups received symptomatic treatment for IPF at this hospital. Among them, only a small number of patients received anti-fibrotic treatments. This highlights the scarcity of treatment methods for this pulmonary fibrosis disease.

In this study, combining univariate and multivariate analyses, we confirmed the following results: (1) Through certain interventions, depression in IPF patients can be effectively controlled within 12 months. If these interventions were not carried out, the severity of depression might gradually increase with the progression of IPF. (2) Among the patients who received depression interventions, the progression of IPF and frailty seemed to remain basically stable to a certain extent and within a certain period of time. In contrast, among the patients who did not receive these interventions, the IPF and frailty seemed to deteriorate significantly within 12 months. This implies that psychological factors occupy a position that cannot be ignored in the evolution process of the IPF and frailty in the patients. Although it was impossible to reverse the progression of IPF by effectively suppressing or eliminating psychological factors, simultaneous physiological and psychological treatment may enable patients to obtain stability for a relatively long time and give them a better chance to live with the disease. In addition, it was worth mentioning that improving depression cannot affect the deterioration of DLCO% pred. A possible explanation was that this lung function index mainly reflected pulmonary ventilation function and had less connection with psychological factors [25].

In recent years, only a few studies have explored the impact of psychological disorders such as depression on lung function. Wang et al.'s cross-sectional study showed that among 352 young college students, both mild and moderate-to-severe depression were associated with a decrease in forced expiratory volume in one second (FEV1). Mild depression was also associated with a decrease in peak expiratory flow. The conclusion was that depression is an independent predictor of decreased FEV1 in young college students [8]. Gunnell et al. conducted a study spanning 11 years on more than 1000 adolescents. The results showed that subjects with more severe initial depressive symptoms had a greater reduction in physical activity in the future [26]. This reduction in activity seems to partially explain the correlation between depression and decreased lung function in adolescent subjects. In addition, Park et al.'s cross-sectional study showed that among 3321 middle-aged and elderly subjects, the FEV1/FVC ratio in the depression group was significantly lower than that in the non-depression group. After adjusting for many confounding factors, in subjects over 50 years old, worsening depression was associated with a lower FEV1 level [9]. The results of this study seem to imply that the correlation between depression and lung function exists simultaneously across different age groups. In conclusion, the results of previous studies are only preliminary, merely revealing the potential relationship between depression and lung function. This study, on the basis of the previous findings, provides more evidence to support the impact of depression on lung function and the protective effect of improving depression on lung function.

Regarding the mechanism of improving depression and then delaying the progression of IPF, we speculated as follows: (1) Physiological mechanism: The improvement of depression can alleviate the immune inflammation and endocrine disorders induced by it, and enhance the body's resistance to combat the progression of IPF [27, 28]. And this study evaluated the fluctuating trends of the peripheral levels of the two inflammatory factors, IL-6 and TNF-α. The results showed that depression intervention could alleviate the continued deterioration of the peripheral levels of these two factors. This seems to partially support the aforementioned physiological mechanism. (2) Psychological mechanism: Many aspects of IPF and frailty should be related to psychological factors. Although a positive psychological state cannot fundamentally reverse the disease, it may give patients a lot of help at the clinical manifestation level or the living level. In addition, patients with mental health might be more actively cooperating with treatment and achieve relatively ideal treatment effects.

This study has the following limitations: (1) The researchers did not directly participate in the intervention of depression. This avoided ethical risks, but it also made it impossible to standardize the intervention measures for different subjects. As a result, this study can only roughly reveal that improving depression may have a protective effect on IPF patients, and the differences in the effectiveness of different intervention measures remain to be explored in future research. (2) Due to practical objective conditions, the follow-up period of this study was only 12 months, which led to an insufficient exploration of the protective effect of antidepressant interventions. In particular, it was impossible to reveal the duration of the effectiveness of these intervention measures and their impact on the patients' survival period. (3) Also limited by objective conditions, this study did not conduct an analysis at the mechanistic level of the above-mentioned correlations or protective effects. We hope that more research can be carried out in the future to address these shortcomings.

In conclusion, this study suggested that antidepressant intervention may, to some extent, delay the deterioration of IPF and frailty in elderly patients within a certain period. Complementing the IPF treatment strategy based on these research findings may potentially benefit elderly IPF patients worldwide in a low-cost and easily implementable manner, which is also in line with the long-term development direction of the bio-psycho-social medical model.

## Supplementary Information

Below is the link to the electronic supplementary material.

Supplementary file1 (DOCX 18 KB)

## Data Availability

Since this study is part of a larger clinical research initiative and other related parallel articles have not yet been published, we are currently not authorized to release these data. Once the entire project is completed, we will promptly make all data publicly available.
