# Supplementary material for: Antidepressant intervention to possibly delay disease progression and frailty in elderly idiopathic pulmonary fibrosis patients: a clinical trial
Source: Aging Clin Exp Res. 2025 Mar 22;37(1):101. doi: 10.1007/s40520-025-03009-4 (PMC11929723; doi:10.1007/s40520-025-03009-4)
Supplement: Supplementary file 1 — Supplementary file1 (DOCX 18 KB) [file 40520_2025_3009_MOESM1_ESM.docx]

**Supplemental Table 1 Baseline depression, idiopathic pulmonary fibrosis, frailty and peripheral inflammation in the two groups**

|  | **Intervention** | **Non-intervention** | **t / χ² value** | **P value** |
| --- | --- | --- | --- | --- |
| Depression |  |  |  |  |
| BDI-II | 23.5±6.5 | 23.3±6.7 | 0.283 | 0.778 |
| Mild (n) | 18 (20.2) | 39 (31.5) | 3.332 | 0.068 |
| Moderate (n) | 60 (67.4) | 69 (55.6) | 3.006 | 0.083 |
| Severe (n) | 11 (12.4) | 16 (12.9) | 0.014 | 0.906 |
| IPF |  |  |  |  |
| Duration (mo) | 5.6±2.2 | 4.5±2.4 | 3.288 | 0.001 |
| FVC %pred | 76.2±11.7 | 78.2±12.2 | 1.194 | 0.234 |
| DLCO %pred | 51.3±9.8 | 52.5±9.8 | 0.886 | 0.377 |
| 6MWT | 396.2±41.8 | 393.0±51.2 | 0.487 | 0.627 |
| mMRC | 2.1±0.9 | 1.9±1.2 | 1.441 | 0.151 |
| Frailty |  |  |  |  |
| CFS | 4.3±1.2 | 4.1±1.2 | 1.136 | 0.257 |
| TFI | 7.5±2.1 | 7.2±2.0 | 1.052 | 0.294 |
| SGRQ | 41.9±14.5 | 38.6±15.9 | 1.575 | 0.117 |
| K-BILD | 69.4±13.7 | 70.6±15.3 | 0.604 | 0.546 |
| Peripheral inflammation |  |  |  |  |
| IL-6 (pg/ml) | 18.42±4.18 | 17.88±4.57 | 0.874 | 0.383 |
| TNF-α (pg/ml) | 35.05±12.04 | 36.00±12.83 | 0.545 | 0.586 |

Note: BDI-II = Beck Depression Inventory-II, IPF = Idiopathic pulmonary fibrosis, FVC %pred = Forced vital capacity percent predicted, DLCO %pred = Diffusing capacity of the lung for carbon monoxide percent predicted, 6MWT = 6-minute walk test, mMRC = modified Medical Research Council dyspnea scale, CFS = Clinical Frailty Scale, TFI = Tilburg Frailty Indicator, SGRQ = St. George's Respiratory Questionnaire, K-BILD = King’s Brief Interstitial Lung Disease questionnaire, IL-6 = Interleukin-6, TNF-α = Tumor necrosis factor-α. Duration indicated the time from the diagnosis of IPF to the current admission. Continuous variables were presented as mean ± standard deviation, and the differences between groups were evaluated by independent sample t-test. Categorical variables were presented as frequency (proportion), and the differences between groups were evaluated by chi-square test. P < 0.05 indicated statistically significant differences.
